# Application of the 3D-MRI on post-operative graft assessment in adolescent patients with ACL reconstruction: A minimal 2-year follow-up

**DOI:** 10.3389/fsurg.2022.1070324

**Published:** 2023-01-06

**Authors:** Xiaona Wang, Yansong Qi, Huricha Bao, Yongsheng Xu

**Affiliations:** ^1^ Department of Imaging Medicine, Inner Mongolia People's Hospital, Hohhot, China; ^2^ Department of Orthopedics (Sports Medicine Center), Inner Mongolia People's Hospital, Hohhot, China

**Keywords:** three-dimensional MRI, adolescent, ACL reconstruction, morphology, cross-sectional area, long-to-short axis ratio, ligamentization

## Abstract

**Background:**

The purpose of the present study was to assess the prognostic morphological changes of the reconstructed hamstring auto-grafts by using reconstructed three-dimensional MRI (3D-MRI) in adolescent patients with ACLR.

**Methods:**

22 adolescent patients (less than 17 years old) were retrospective included between January 1, 2018, and October 31, 2020, in our department. The patients were divided into 2 subgroups: subgroup A (<14 years old) and subgroup B (≥14 years old). 3D-MRI was used to detect the total cross-sectional area (TCA) and long-to-short axis (LSA) ratio of the reconstructed ACL graft at the proximal, mid-point, and distal regions. The minimal follow-up was 2 years.

**Results:**

The averaged follow-up of subgroup A and B was 37.8 ± 5.6 and 37.6 ± 6.5 months, respectively. Comparing to the initial graft (ACLR operation), the TCA of reconstructed ACL was increased by 30.6% on average, and the TCAs at proximal, mid-point, and distal regions were increased by 56.4%, 50.0%, and 17.7%, respectively, inner-group comparisons showed that the TCAs of the 3 region in subgroup A were all increased at the follow-up (*P *= 0.002) (*P *< 0.001) (*P *< 0.001), however, only increased mid-point (*P *= 0.024) and distal TCAs (*P *< 0.001) were found in subgroup B. Comparing to the native ACL, the proximal LSA ratio in subgroup A was comparable, while it was lower in subgroup B than the native ACL (*P *= 0.004), the distal LSA ratios in the 2 subgroups were both lower than the native ACL (*P *= 0.004) (*P *= 0.006).

**Conclusions:**

3D-MRI assessment can exactly identify the morphological changes of the graft in adolescent patients with ACLR, the TCA of the constructed ACL was increased compared to the initial graft, however, the LSA ratio was still lower than the native ACL. Younger adolescent patients may have a better potential on the ligamentization after ACLR than the older adolescent patients.

## Introduction

In recent years, the incidence of anterior cruciate ligament (ACL) rupture is progressing at a greater rate in the pediatric and adolescent population, in comparison with adults (1–3). Considerable attention needs to be paid on the treatment of ACL rupture in this skeletally immature adolescent population. ACL reconstruction (ACLR) has become the gold standard of treatment for the adolescent population seeking to return to sports (4). The hamstring tendon auto-graft is preferred in the ACLR for adolescent patients, which avoids the damage from open epiphysis by the bone-patellar tendon-bone (BPTB) graft (5, 6). Young age, the smaller size of the auto-graft, and higher activity level were considered to be the risk factors related to ACL re-rupture and clinical failures in adolescent patients (7, 8).

Reconstructed three-dimensional MRI (3D-MRI) is a new objective imaging assessment for ALCR (9). The latest study has reported that using 3D-MRI on assessment of the reconstructed ACL showed a substantial intra- and inter-observer agreement (10). It has also been shown that 3D-MRI identification of the reconstructed ACL compares favorably and can be used interchangeably with the anatomic identification of cadaveric ACL (11). Given that prediction of the diameter of the semitendinosus and gracilis tendon from adolescent patients may represent insufficient graft strength (12), the objective assessments of the reconstructed ACL tendon in post-operation are of great significance to clinical practice.

Although several different ACLR techniques are available for skeletally immature patients, however, the optimal surgical technique for ACLR in younger children is still controversial at present (13). One of the main reasons is lacking the objective imaging assessments of the reconstructed ACL tendon in post-operation follow-ups. The purpose of this study was to assess the prognostic morphological changes of the reconstructed hamstring auto-grafts by using 3D-MRI in adolescent patients with ACLR.

## Methods

### Patient involvement

This was a self-control clinical study. Adolescent patients diagnosed with ACL rupture were included between January 1, 2018, and October 31, 2020, in our department. The inclusion criteria: (1) patients younger than 17 years old; (2) with open physis diagnosed through knee radiographs images at the time of surgery (14); (3) single-bundle trans-physeal ACLR with hamstring grafts; (4) follow-up more than 2 years. The exclusion criteria: (1) multi-ligament injury; (2) ACL re-rupture; (3) history of lower extremity fracture, ligament rupture, and operations; (4) systematic diseases, such as rheumatoid arthritis, and motor-nerve system diseases. The included patients were recruited retrospectively with the recorded contact information. The patients were divided into 2 subgroups: subgroup A (patients < 14 years old) and subgroup B (patients ≥ 14 years old).

Ethics approval was obtained before the enrolment of patients into the study. Our study was conducted following the international ethical standards required for publication (15).

### ALCR operation

The trans-physeal reconstruction techniques using autologous single-bundle hamstring grafts were applied to all the patients by the senior author.

The procedure was performed under combined spinal-epidural anesthesia with the arthroscopic assessment, which allowed the treatment of associated injuries. Meniscal lesions were either sutured or resected. After harvesting the semitendinosus and gracilis, tendons were cleaned of muscle insertion. Grafts were tightened and half-folded to be prepared for insertion into the patient's knee (16). Before implantation, a circular millimeter ruler was used to measure the diameter of the graft at its middle point, which will be located inside the joint. During the surgical procedure, the positioning of the femoral and tibial tunnel was based on the anatomic center of the native ACL footprint after removing the remnant (17). According to the size of previously measured grafts, the tibial and femoral tunnels were reamed through the central epiphysis independently. Afterward, the four-strand semitendinosus and gracilis tendon was taken care of to fill in both tunnels. On the femoral side, standard far-posterior tunnel placement was undertaken with suspensory fixation using an EndobuttonCL (Smith & Nephew). Double staples were used for tibial fixation. All 22 patients were rehabilitated with braces according to the same guidelines by physical therapists, emphasizing early full extension and range of motion.

### Follow-ups

The follow-up started when the ACLR was completed. The end was re-rupture/death/missing, whichever occurred first. The minimum follow-up was 2 year (y). General characteristics included: sex, age, surgery duration (the injury time before the operation), and follow-up time, other clinical information included: associated injuries (meniscus tear, cartilage, and other injuries) and complications. The clinical assessments of the outcomes of ACLR included: knee 3D-MRI, as well as the subjective scoring systems of knee function, which were performed at 1 and 2y follow-ups. All of the follow-up data was checked and entered into the database by two independent researchers, and a double-entry is also carried out for control of quality.

### 3D-MRI Assessment

The 3D-MRI was performed by a radiologist with 5 years of experience in knee MRI images. At the follow-ups, 3D-MRI was utilized for bilateral knees with 1-mm slice thickness T2 weighted images with no fat suppression (GE, 3.0 T Signa HDxt at resolution 512*512 pixels bitmap). The images were introduced into AW Server 2.0 (GE Health Care) to reconstruct three-dimensional models. A reference line passing through the geometric centroid of the entire graft was identified.

### Graft size assessment

**Figure 1:**
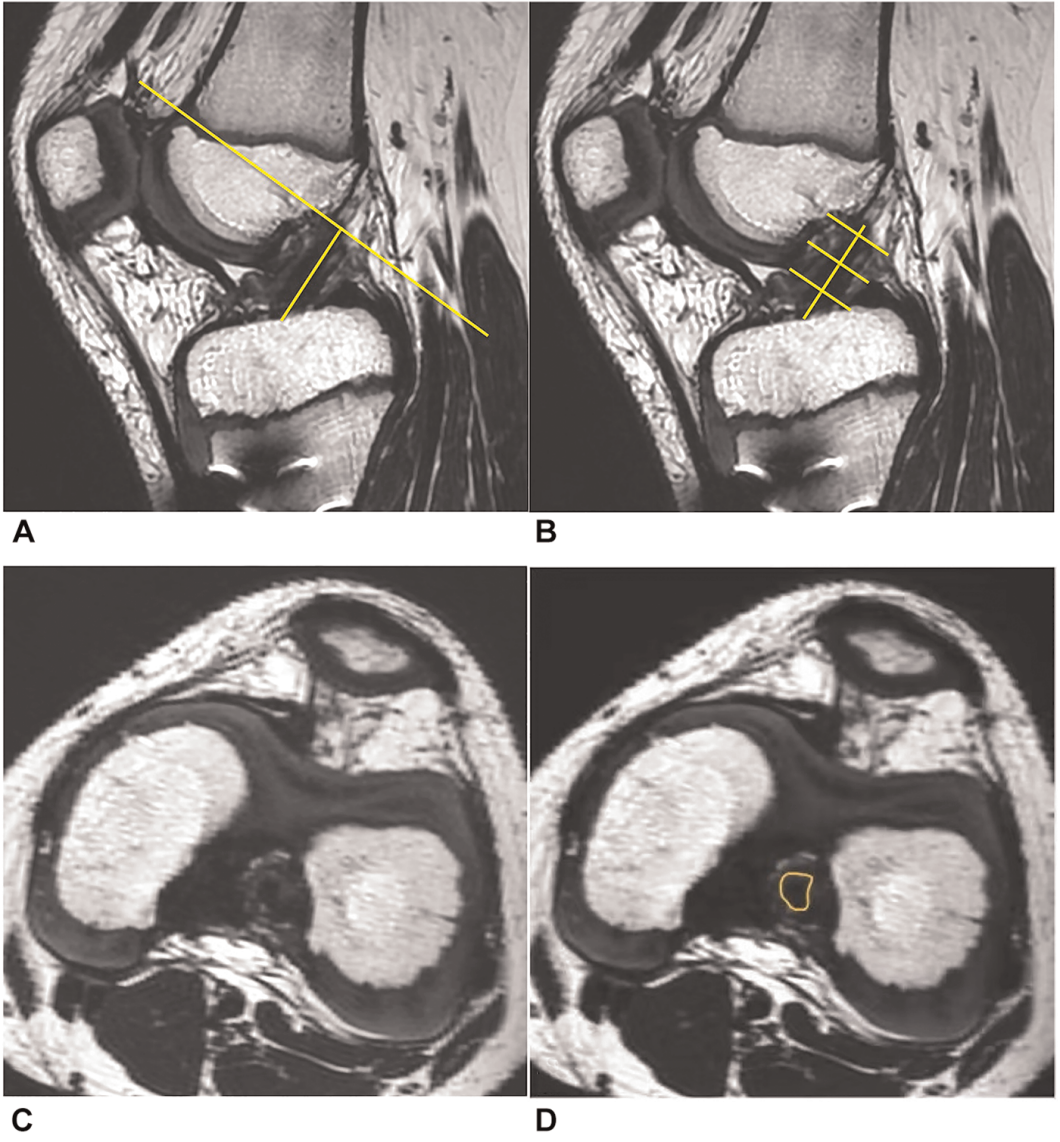
3D-MRI assessment of the ACL. (**A)** A reference line across the center of the entire ACL graft and a perpendicular plane. (**B**) The 2nd, 3rd, and 4th slices represented three evenly spaced cross-sectional slices (at 25%, 50%, and 75% of the ligament's overall length from the femoral attachment to the tibial attachment). (**C**) Cross-section of ACL graft at the mid-point location. (**D**) Outline of the cross-sectional area.

Grafts measurements in 3D-MRI were performed separately by 2 researchers (a radiologist with 5 years of experience in knee MRI images and a sports medicine doctor with more than 10 years of clinical experience). Perpendicular planes to the central axis of ACL were marked at 25% (proximal), 50% (mid-point), and 75% (distal) of the ligament's overall length (from the femoral attachment to the tibial attachment), to create 3 cross-sectional slices (Figures 1A,B). The total cross-sectional area (TCA) was calculated, TCA = π*(averaged diameter of the 3 slices/2)^2^. The TCA provides information about the size of the graft, which could be compared to the initial size recorded during surgery. At each cross-section slice, outline of the cross-sectional area of ACL graft was carefully identified to extract the LSA (Figures 1C,D), and the long-to-short-axis (LSA) ratio was calculated. LSA ratio provides insight into the shape of the transverse section, whereby a ratio of 1:1 implies a round ligament.

To compare those parameters of the ACL graft with the native ACL, the same procedure was applied to the contralateral knee at the same time point.

### Statistical analysis

Continuous data were expressed as mean ± SD, and the comparisons were processed by the paired *t-*tests and Levene variance homogeneity tests. Count data were expressed as number (n) and rate (/), and comparisons of the count data were processed by the Chi-square test or Fisher's exact test. The level of significance was set at 0.05. All of the statistical analyses were performed using SPSS 20.0 (SPSS Inc., 2009, Chicago, IL, USA).

## Results

### Basic characteristics

Finally, 22 adolescent patients meeting all the criteria were included. Subgroup A (<14 years old) consisted of 9 patients: 5 suffered a sprain of the knee when doing competitive sports, 4 sprained the knee by themselves when doing un-competitive sports, such as skiing and skating; subgroup B (≥14 years old) consisted of 13 patients: 6 suffered a sprain of the knee when doing competitive sports, 3 sprained the knee by themselves when doing un-competitive sports, 2 slipped and sprained the knee by themselves during daily routine, and 2 were caused by vehicle accidents. The general characteristics and clinical information of the 2 subgroups were listed below (Table 1), and no complication of abnormal bone growth or angular deformity was observed during the follow-up period.

**Table 1:** Basic characteristics of the subgroups in adolescent patients with ACLR.

Characteristics	Subgroup A (<14 years old)	Subgroup B (≥14 years old)
Enrolled subjects (*n*)	9	13
Sex (male/female)	7/2	8/5
Age (year)	12.0 ± 1.1	15.7 ± 1.2
BMI	22.17 ± 1.23	22.25 ± 1.51
Surgery duration (week)	2.1 ± 2.9	3.7 ± 4.5
Meniscus injury (with/without)	1/8	2/11
MCL injury (with/without)	1/8	1/12
Graft diameter (mm)	7.86 ± 0.23	7.82 ± 0.25
Follow-up time (month)	37.8 ± 5.6	37.6 ± 6.5

Note: BMI, Body Mass Index; ACLR, anterior cruciate ligament reconstruction; MCL, medial collateral ligament.

### TCA comparison between the initial graft and follow-up

**Figure 2:**
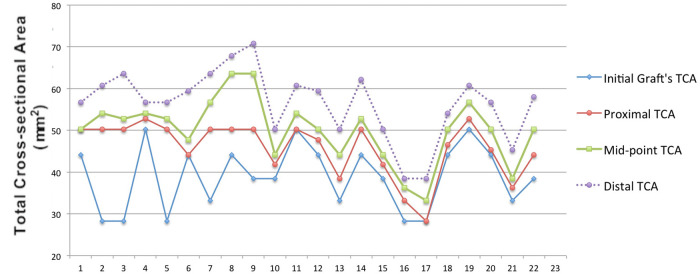
Self-control comparisons of TCA between the initial graft and follow-up in each patient the largest growths were found in the distal TCAs (blue) in all patients, and then the mid-point (green) and distal (Red) TCAs, respectively.

The sizes of the ACL grafts were increased at the 3 slices of 3D-MRI at the follow-up in all of the patients (Figure 2), and the TCA at proximal, mid-point, and distal regions were increased by 56.4%, 50.0%, and 17.7%, respectively. The maximal increase was observed in the proximal region, while the increase in the distal region was relatively less. Averagely, the TCA of ACL graft was increased by 30.6% in post-operation at a minimal 2y follow-up.

Inner-group comparisons of Subgroup A showed that the TCA of reconstructed ACL at the proximal, mid-point, and distal regions were increased at minimal 2y follow-up compared to the initial state at ACLR operation, however, only increased TCA of reconstructed ACL at the mid-point and distal regions were found in Subgroup B at minimal 2y follow-up compared to the initial state (Table 2).

**Table 2:** TCA comparisons between the initial graft and follow-up in the 2 subgroups.

TCA	Subgroup A (<14 years old)			Subgroup B (≥14 years old)		
	Initial Graft	Reconstructed ACL	*P* value	Initial Graft	Reconstructed ACL	*P* value
Proximal TCA (mm^2^)	37.68 ± 8.45	49.85 ± 2.29	*t *= −4.107 *P *= 0.002**	39.64 ± 7.38	42.84 ± 7.21	*t *= −1.117 *P* = 0.275
Mid-point TCA (mm^2^)	37.68 ± 8.45	55.07 ± 5.44	*t *= −5.191 *P *< 0.001**	39.64 ± 7.38	46.53 ± 7.19	*t *= −2.412 *P* = 0.024*
Distal TCA (mm^2^)	37.68 ± 8.45	61.81 ± 5.12	*t *= −7.329 *P *< 0.001**	39.64 ± 7.38	52.69 ± 8.11	*t *= −4.291 *P *< 0.001**

Note: *P* < 0.01.

** *P* < 0.05.

* Total cross-sectional area (TCA).

### LSA comparison between the reconstructed ACL and native ACL

Inner-group comparisons showed that the proximal LSA ratio of reconstructed ACL in subgroup A was comparable with the native ACL on the healthy contralateral side, while the proximal LSA ratio of reconstructed ACL in subgroup B was lower than that of the native ACL; the mid-point LSA ratio of reconstructed ACL did not have significant difference with the native ACL in the 2 subgroups; the distal LSA ratios of reconstructed ACL in the 2 subgroups were lower than that of the native ACL (Table 3).

**Table 3:** LSA comparisons between the reconstructed ACL and native ACL at follow-up.

LSA	Subgroup A (<14 years old)			Subgroup B (≥14 years old)		
	Native ACL	Reconstructed ACL	*P* value	Native ACL	Reconstructed ACL	*P* value
Proximal LSA ratio	1.29 ± 0.10	1.39 ± 0.16	*t *= −1.634 *P *= 0.122	1.21 ± 0.18	1.43 ± 0.17	*t *= −3.138 *P *= 0.004**
Mid-point LSA ratio	1.67 ± 0.13	1.54 ± 0.14	*t *= 2.028 *P *= 0.060	1.60 ± 0.14	1.61 ± 0.17	*t *= −0.173 *P* = 0.864
Distal LSA ratio	2.59 ± 0.17	2.07 ± 0.39	*t *= 3.683 *P *= 0.004**	2.52 ± 0.16	2.10 ± 0.45	*t *= 3.218 *P *= 0.006**

Note: *P* < 0.01.

** Long-to-short-axis.

**Table 4:** Motor function comparisons between the subgroups at the follow-up.

Motor function	Subgroup A (< 14 years old)	Subgroup B (≫ 14 years old)	*P* value
			*t* = 1.184
Lysholm	86.22 ± 2.91	84.31 ± 4.19	
			*P* = 0.250
			*t* = 1.774
IKDC	87.22 ± 4.18	84.69 ± 2.53	
			*P* = 0.091
	yes 9	yes 13	
Return to sports			*P* = 1.000
	no 0	no 0	

## Discussion

It has been reported the incidence of ACL rupture in adolescents has increased with a peak at 17 years old (18). Since ACLR results in higher joint stability and lower risks of additional meniscal and chondral injuries in adolescent patients, compared to non-operative or delayed operative treatment (4), ACLR has become the gold standard of treatment for adolescent patients. Objective imaging assessments of the reconstructed ACL tendon in post-operation follow-ups were relatively rare in present studies. As the morphology parameters of the reconstructed ACL are closely associated with the function and outcome, for example, the TCA and LSA ratio, the 3D-MRI assessment is of great significance to clinical practice.

The present study found that the 3D-MRI assessment can exactly identify the morphological changes of the graft in adolescent patients. Most adolescents become skeletally mature over 17 years old (19). Our results showed that compared with the initial size at surgery, the TCA of the graft based on 3D-MRI was shown to increase by 30.6% on average at a minimal 2y follow-up in this population. The increasing percentage of TCA at three slices from proximal, mid-point and distal regions were increased by 56.4%, 50.0%, and 17.7%, respectively. The TCA provides information about graft size. It has been proved that the TCA of graft measured by MRI in post-operation was well correlated with those directly measured during the ACLR operation (20). In order to assess the size changes of the reconstructed ACL in long-term prognosis, we compared the TCA measured by 3D-MRIs at the follow-up with the data of graft measured during the ACLR operation. Similar to our results, Min et al. also showed that the diameter of the autologous patellar tendon graft increased by 70% in 23 patients, which was measured in an oblique axial image directly scanned with 1.5 T MR (21). Hamada et al. showed that measured in an oblique coronal ACL image, the graft cross-sectional area increased by 29% at 12 months after surgery (20). The increasing size of the ACL graft in this study could be attributed to the process of ligamentization (22), during which the graft underwent remodeling and became hypertrophy. Our study suggests that the 3D-MRI assessment can exactly identify the morphological changes of the graft in adolescent patients. The present study also found a difference in the graft ligamentization process between the 2 subgroups based on the different age distribution. Our results showed that compared to the initial graft, the proximal, mid-point, and distal TCA of reconstructed ACL were all increased in patients < 14 years old, while only the mid-point and distal TCA were increased in patients ≥ 14 years old, which suggested that adolescent patient with younger age may have a better potential on ligamentization after ACLR.

The LSA ratio is another morphological parameter extracted from the 3D-MRI, which indicates the transverse section shape of the graft, and a ratio of 1:1 implies a cylindrical ligament. Our results showed that the proximal, mid-point and distal LSA ratios were beyond the initial ratio (1:1, at the ALCR operation), what's more, were gradually increased. It suggests that LSA ratios measured in 3D-MRI demonstrate that the cross-sectional shape of the intra-articular graft has changed from the original cylindrical shape to a fan-shaped flat ligament, and 3D-MRI assessment can exactly identify the morphological changes in the graft's ligamentization process in adolescent patients. Our results found that the LSAs of the constructed ACL in the 2 subgroups were lower than the native ACL, which suggested the constructed ACL was prone to cylindrical compared to the native ACL with a flatter fan-shaped. It has been reported that the constructed ACL was more cylindrical compared with the native ACL (9, 23), and a cylindrical ACL may cause impingement on PCL and femoral notch (9, 24). Therefore, the double-bundle ACLR with a more similar shape to the native ACL can avoid those disadvantages (25), and 3D-MRI has the benefit of assessing the morphological characteristics of post-operative ligamentization in adolescent patients. In addition, the present study also found a difference in the LSA between the 2 subgroups. Our results showed that the proximal LSA ratio of reconstructed ACL in patients < 14 years old was comparable with the native ACL, while the proximal LSA ratio of reconstructed ACL in patients ≥ 14 years old was higher than that of the native ACL. The proximal LSA ratio indicates the transverse section shape of the foot-print region of the graft. Our results suggested that adolescent patients with younger age can result in a more similar footprint shape to the native ACL.

The limitations of our study were as follows: (1) this was a retrospective study, and the relatively small sample size and variable follow-up periods might result in bias of the results, especially for the results based on two subgroups; (2) lack of observation on the dynamic changes of the ACL graft in post-operation. Further longitudinal studies with more samples and follow-up points are required to explore more valuable morphology changes in adolescent patients with ACLR.

## Conclusion

3D-MRI assessment can exactly identify the morphological changes of the graft in adolescent patients with ACLR, the TCA of the constructed ACL was increased compared to the initial graft, however, the LSA of the constructed ACL was still lower than the native ACL at a minimal 2y follow-up. Younger adolescent patients (<14 years old) may have a better potential on the ligamentization after ACLR than the older adolescent patients (≥14 years old).

## Data Availability

The original contributions presented in the study are included in the article/Supplementary Material, further inquiries can be directed to the corresponding author/s.
